# Physical Activity and the Development of Substance Use Disorders: Current Knowledge and Future Directions

**DOI:** 10.1097/pp9.0000000000000018

**Published:** 2018-05-11

**Authors:** Angelique G. Brellenthin, Duck-chul Lee

**Affiliations:** Department of Kinesiology, College of Human Sciences, Iowa State University, Ames, IA

**Keywords:** physical activity, substance use, alcohol, tobacco, marijuana, illicit drug, mechanistic pathway

## Abstract

Physical activity and exercise are positive health behaviors that have been shown to reduce the risk of physical and psychological diseases. There is a strong rationale that physical activity could be a protective factor against the development of substance use disorders (SUDs), which include some of the most common mental health conditions such as tobacco and alcohol use disorder. This review examined the epidemiological literature to describe the associations of physical activity and substance use across the lifespan. The findings indicated that physical activity is positively associated with current and future alcohol use but negatively associated with tobacco and other drug use, with the strongest support originating from adolescent and young adult samples. Considerably less data exist on physical activity and other drug use in later life. Limitations in previous studies, such as the indeterminate measurement of physical activity and absence of clinical substance use disorder endpoints, should be addressed in future investigations to provide clarity regarding the strength and directions of these relationships among different substances and populations.

## Significance of substance use disorders and role of physical activity

Substance use disorders (SUDs) are mental health disorders that occur when the repeated use of a substance (eg, alcohol, tobacco, drugs) causes clinically significant psychophysical distress and impairment. The *Diagnostic and Statistical Manual of Mental Disorders*, Fifth Edition (DSM-5) criteria for SUDs include increased use over time, craving, tolerance, and social harm. SUDs are among the leading causes of premature mortality in the United States, contributing to an estimated 22% of preventable deaths.^[1]^ The economic burden of SUDs is estimated to approach $740 billion annually through reduced labor and workforce productivity and increased criminal, legal, and healthcare costs related to both the primary SUD and comorbid health problems (eg, cardiovascular and infectious diseases).^[2]^ SUDs are also among the most common mental health disorders besides depression and anxiety, with an estimated 29%, 28%, and 10% of adults having an alcohol, tobacco, or other drug use disorder, respectively, at some point during their lives.^[3–5]^ Less than 1 in 5 individuals with lifetime SUD will receive treatment,^[5]^ and 40–60% of those treated for SUDs will relapse within 1 year.^[6]^ SUDs are characterized as chronic, relapsing disorders with a distinct neurobiological disease progression,^[7]^ and it is not clear at this time whether the brain can fully restore itself to its preabuse state.^[8,9]^ Thus, like many other chronic diseases, the most effective public health strategy for minimizing the societal and individual impact of SUDs is preventing them in the first place.

**Figure 1.**
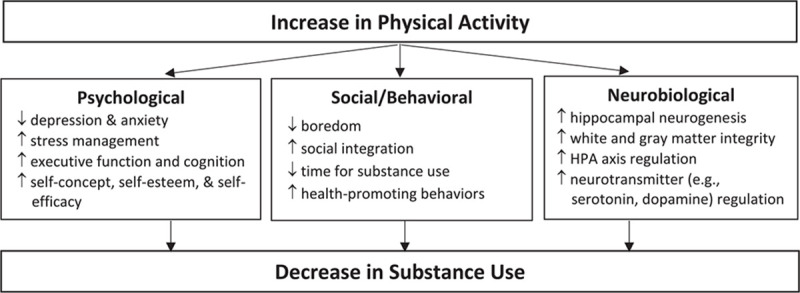
Potential mechanistic pathway between physical activity and substance use. ↑Indicates an increase; ↓ indicates a decrease. Support for the proposed psychological and social/behavioral mechanisms is primarily from human research while support for the neurobiological mechanisms is primarily from animal research. HPA, hypothalamic-pituitary-adrenal.

Physical activity (any bodily movement produced by the skeletal muscles) and exercise (a subset of physical activity that involves planned, structured activity sessions for the purposes of improving health and fitness) are positive health behaviors that have been shown to prevent and treat a multitude of physical and mental health conditions.^[10]^ The development of SUDs is a multifactorial process involving genetic, environmental, and psychosocial elements. Physical activity has the ability to influence psychological, behavioral, and neurochemical processes that contribute to SUDs. Broadly, health-promoting behaviors like physical activity tend to cluster together as part of a healthy lifestyle. Physically active individuals tend to have lower body mass indexes, do not smoke cigarettes, and drink alcohol in moderation,^[11]^ which has been associated with reduced all-cause and cardiovascular disease mortality.^[12]^ Exercise can prevent and treat mental health conditions such as depression and anxiety,^[13]^ which are highly comorbid with SUDs.^[14]^ Many forms of exercise also have a social component promoting increased societal integration and preventing loneliness and isolation, which have been associated with increased substance use.^[15]^ Finally, there is substantial preclinical evidence demonstrating that physical activity reduces rates of drug use acquisition and escalation and rates of reinstatement of drug-seeking behaviors in a variety of animal experiments.^[16,17]^ Fig 1 illustrates the potential mechanisms underlying the relationship between physical activity and substance use as suggested by preclinical and clinical studies; however, large prospective epidemiological studies and controlled clinical trials are clearly warranted to verify these relationships.

Given the considerable preclinical evidence in animals and the rationale that regular physical activity could be a protective factor against the development of SUDs, the purpose of this review is to summarize the epidemiological literature in humans examining physical activity and the prevalence and incidence of SUDs across the lifespan. Furthermore, because of limited data existing on physical activity and clinical SUD diagnoses, the majority of studies reviewed focus on physical activity and amounts of substance use (eg, alcoholic drinks/wk) rather than DSM-defined SUDs. The evidence regarding exercise as a treatment for SUDs will not be addressed, but refer to earlier studies for detailed reviews of this literature.^[16,18,19]^

## Adolescents (approximately 10–17 years old)

Most substance use prevention efforts focus on adolescence, since substance use during this developmental period is a significant predictor of substance use later in life.^[20]^ The majority of adults with clinical SUDs report their first substance use before the age of 18 years.^[21]^ The effects of substance use during adolescence are also more severe than the effects of drug use during other developmental periods. The highly plastic and actively maturing adolescent brain is more susceptible to the neurochemical effects of drugs, which can result in irreversible damage to many cognitive functions including memory, decision-making, motivation, and impulsivity.^[7,22]^ Thus, drug use during adolescence can impact the life course of an individual as it is associated with lower lifetime earnings,^[23]^ greater incidence of comorbid mental and physical health conditions,^[24]^ and premature mortality.^[25]^

In general, cross-sectional studies conducted in junior high and high school students have found that physical activity and exercise are inversely associated with cigarette smoking and illicit drug use but positively associated with alcohol use and binge drinking behaviors.^[26,27]^ In fact, 1 study conducted among 13,318 Canadian adolescents estimated that nearly 13% of binge drinking in boys and 8% of binge drinking in girls could be prevented if they did not participate in team sports.^[28]^ Conversely, the authors found that current cigarette smoking would be 22–23% higher in boys and girls if they did not participate in team sports.^[28]^ In addition to cross-sectional studies, Kwan et al.^[29]^ systematically reviewed 17 longitudinal studies and found that sports participation was associated with increased future alcohol use but decreased illicit drug use (other than marijuana) in the majority of studies. Studies that investigated sports participation and marijuana use had mixed results, with some reporting no relationship between sports participation and marijuana use and others reporting both positive and negative associations between sports participation and marijuana use.^[29]^

A problem with quantifying physical activity solely through athletic participation is that there is considerable heterogeneity in the actual levels of physical activity across different sports. For example, greater amounts of exercise, independent of sports team participation, have been associated with less cigarette and illicit drug use but also with reduced alcohol use and frequency of getting drunk.^[30,31]^ The social and competitive elements also differ greatly between sports, which may influence the relationship between physical activity and substance use. For instance, participation in team sports appears to be more strongly associated with alcohol use, whereas participation in individual or endurance sports is associated with lower use of all substances including alcohol.^[28,32]^

Few intervention studies have been conducted that can expand upon the findings of observational studies. Collingwood et al.^[33]^ found that a 12-week program, which emphasized physical activity and fitness as part of a drug prevention effort, decreased the percentage of adolescents who used cigarettes while improving protective factors such as mood and self-concept. However, this study lacked a control group and had a low number (approximately 13% of sample) of substance using adolescents at baseline.^[33]^ No other study has directly assessed the effects of an exercise program on the prevention or escalation of substance use, which limits inferences into causality between physical activity and substance use among high school youth.

There are equivocal findings regarding the lasting impact of high school sports and exercise participation into young adulthood. In a study of 4,240 individual twins over 6–8 years, Korhonen et al.^[34]^ found that consistently inactive individuals (engaging in exercise ≤ 1–2 times/mo) had twice the odds of screening positive for risky alcohol use and 3.75 times increased odds of using illicit drugs compared with consistently active individuals (exercising ≥ 4–5 times/wk).^[34]^ However, physical activity defined through sports participation continues to produce mixed findings. Green et al.^[35]^ analyzed data from 24,799 college students and found that participation in organized athletics during high school and college was associated with higher rates of binge drinking. Binge drinking increased when individuals became involved in college sports even if they had not been active in high school. Conversely, binge drinking behaviors persisted into college, rather than declined, even when athletic participation ended with high school.^[35]^

Finally, findings are also mixed when substance use outcomes are defined in terms of a clinically diagnosed SUD as opposed to increased rates of use. Ströhle et al.^[36]^ found that among 2,548 adolescents, those who were regularly active (exercising daily or several times/wk) had a reduced prevalence of having any SUD [odds ratio (OR) = 0.60 (95% confidence interval (95% CI) = 0.47–0.76)] in the past 12 months compared with adolescents who were inactive (exercising ≤ 1 time/mo). However, in a 4-year follow-up analysis of the same sample, there were no significant differences between inactive and regularly active adolescents in the incidence of any SUD, but individuals who were irregularly active (exercising 1–4 times/mo) had a reduced risk of incident SUD compared with inactive adolescents [OR = 0.48 (95% CI = 0.31–0.75)].^[36]^ Conversely, Veliz and McCabe^[37]^ found that among 4,187 college students, individuals who had participated in high school sports had a 50% increased prevalence of alcohol use disorder in college than nonsports participants [OR = 1.52 (95% CI = 1.22–1.89)]. There were no significant findings between high school sports team participation and other drug use disorders in college.^[37]^ Likewise, Suetani et al.^[38]^ found that among 3,493 adolescents, those who engaged in infrequent physical activity (exercise or sports 1–3 days/wk) at baseline had a reduced odds [OR = 0.75 (95% CI = 0.62–0.91)] of developing any SUD at 7-year follow-up compared with those reporting frequent physical activity (exercise or sports ≥4 days/wk).^[38]^ In summary, physical activity in adolescents, typically measured through sports team participation, is associated with reduced cigarette and illicit drug use but increased alcohol use. At this time, there is not a clear relationship between physical activity during adolescence and the development of a clinical SUD in young adulthood.

## Young adults (approximately 18–34 years old)

Young adulthood is another time period during which individuals are susceptible to developing an SUD. Not only is the brain continuing to develop throughout young adulthood, but also many substances become dramatically more accessible as a result of surpassing age restrictions for legal use (eg, 18 years for tobacco, 21 years for alcohol in the United States). Additionally, there is less parental supervision and a considerable shift in the individual’s routine and environment as they leave home to attend college or work.

Young adults have the highest rates of substance use compared with any other age group. According to the 2014 US National Survey on Drug Use and Health, 86% of college-aged students have consumed alcohol at least once in their lives, and 38% of college students have engaged in binge drinking in the past month.^[39]^ By the end of college, 68% of students will have used marijuana, and 35% will have used prescription stimulants (eg, Ritalin or Adderall) for nonmedical reasons.^[40]^ College students first try illicit drugs (other than marijuana) in college rather than in high school, suggesting that young adults may be the most vulnerable to developing an illicit drug use disorder.^[40]^ Furthermore, early drug use appears to predict long-term health complications. Kertesz et al.^[41]^ found that illicit drug use during young adulthood was associated with a decline in self-reported health over a period of 15 years after controlling for several covariates including physical activity, which was inversely associated with health decline.^[41]^ In addition to poor health in later life, illicit drug use has been associated with an increased risk of death [hazard ratio (HR) =2.40 (95% CI = 1.65–3.48) for heroin; HR = 1.27 (95% CI = 1.04–1.55) for cocaine] over a 20-year period even after controlling for other risk factors including physical activity, smoking, and alcohol use.^[25]^ Thus, there is an urgent need to explore SUD prevention strategies, including physical activity and exercise, in young adulthood.

Exercise participation during college is typically thought of as a way to promote healthy habits and minimize risky behaviors; however, the relationship between exercise participation and substance use among young adults is not clear. Dunn and Wang^[42]^ found that among 2,436 college students (18–23 years old), nonactive students (no exercise/wk) were at an increased risk of being regular smokers [OR = 1.50 (95% CI = 1.03–1.91) for females; OR = 1.84 (95% CI = 2.00–2.78) for males] compared with active students (exercising ≥ 3 times/wk). However, active students were more likely to binge drink in the past 30 days compared with nonactive students [OR = 1.56 (95% CI = 1.20–2.02) for females; OR = 1.76 (95% CI = 1.25–2.46) for males]. Furthermore, active females were less likely than nonactive females to have used marijuana [OR = 0.72 (95% CI = 0.45–0.91)] or cocaine [OR = 0.98 (95% CI = 0.97–0.99)] in the past 30 days. There were no significant associations between levels of activity and other illicit drug use in males.^[42]^ Similar results were found by Moore and Werch^[43]^, in that college students who participated in frequent vigorous exercise (5–7 days/wk) drank significantly more often, consumed greater amounts of alcohol, and binge drank more often compared with infrequent exercisers (0–2 days/wk). Conversely, frequent exercisers had lower cigarette use than infrequent exercisers, though there was no association between exercise frequency and marijuana use.^[43]^ Henchoz et al.^[44]^ examined the longitudinal associations between baseline physical activity, exercise, and sports participation and substance use in 5,223 young adult Swiss men over 15 months. Regular sport and exercise participation was associated with a reduced prevalence of cigarette use [OR = 0.63 (95% CI = 0.46–0.85)] and cannabis use [OR = 0.60 (95% CI = 0.40–0.89)], but had no association with alcohol abuse at follow-up. When controlling for leisure-time sport and exercise, occupational physical activity was associated with an increased prevalence of cannabis use [OR = 1.78 (95% CI = 1.01–3.12)], though sport and exercise continued to be associated with lower cigarette and cannabis use when controlling for occupational physical activity.^[44]^ These results suggest that occupational physical activity (eg, manual labor) is associated with increased substance use while leisure-time physical activity participation is not. Lisha and Sussman^[45]^ reviewed 34 studies examining the relationship between sports team participation in both high school (n = 9 studies) and college (n = 24 studies), and summarized that being on a sports team was associated with increased alcohol use but reduced cigarette and illicit drug use (with equivocal findings regarding marijuana).^[45]^

Overall, the findings in high school and college-aged youth indicate a mixed relationship between physical activity and substance use. Physical activity appears to be inversely associated with cigarette and illicit substance use but positively associated with alcohol use. More research is warranted examining the long-term repercussions of these relationships. For instance, it is possible that the potential benefits of preventing cigarette smoking through physical activity and sports participation outweigh the risks of increased alcohol use, due to smoking’s strong detrimental associations with future morbidity and mortality. In addition, there is a nonlinear relationship between alcohol consumption and all-cause and cardiovascular mortality, with moderate drinking being protective against premature mortality compared with abstinence or heavy drinking.^[12]^ It is crucial to determine how physical activity behaviors and drinking patterns change from young adulthood to middle age and how their interaction influences health over time.

## Adults (approximately 35–54 years old)

Substance use is a public health concern that persists into middle age. In 2016, approximately 48% of U.S. adults age 35 years and older have used at least 1 illicit drug in their lifetime, and 12% have used an illicit drug in the past year. In the past month, 23% of adults have used tobacco products, 21% have engaged in binge drinking, and 5% drank heavily (binge drinking ≥5 days during the past 30 days).^[46]^ Despite high rates of substance use among adults, SUD prevention efforts do not heavily focus on the general adult population because most adults initiate substance use before entering adulthood. For example, 87% of daily smokers began smoking before the age of 18, and 99% began before the age of 30.^[21]^ Alarmingly, opioids (eg, heroin and prescription pain relievers) are the most commonly abused substances among adults who initiate drug use after 25 years.^[21]^

Despite the more heavily researched areas of physical activity and substance use in adolescents and young adults, substantially fewer data exist on the relationship between exercise and SUDs in the general adult population. Broadly, there are numerous reports that physical activity is inversely associated with tobacco use in adults.^[47]^ Though tobacco use often tracks with illicit drug use in adolescent populations, there is scant evidence regarding the effects of physical activity on both tobacco and illicit drug use in adults. A cross-sectional analysis of 8,098 U.S. adults found that physically active respondents (responding that they exercised “regularly” as opposed to “never” or “occasionally”) were less likely to have several mental health conditions, including depression or anxiety, but were just as likely to have substance use dependence (as defined by the DSM-IV) as inactive respondents.^[48]^ Likewise, in 1 study that specifically examined resistance exercise training and drug use, Molero et al.^[49]^ found no relationship between the frequency of weekly weight training and rates of illicit drug use among 1,969 gym attendees in Sweden.^[49]^ Although there is a dearth of evidence examining physical activity and opioid use specifically, there is considerable evidence demonstrating that exercise has hypoalgesic effects (ie, it elicits a reduction in pain sensation during and following exercise).^[50]^ Physically active adults are also better able to modulate their pain than physically inactive adults.^[51]^ These findings may suggest that physical activity could protect against future opioid abuse, but more research is clearly needed.

Most research in the general adult population has focused on physical activity and alcohol consumption, and the preponderance of evidence suggests that physically active adults are also more likely to be moderate drinkers (eg, ≤7 drinks/wk for women; ≤14 drinks/wk for men).^[52]^ However, when alcohol use is defined as “heavy” or “hazardous,” there are mixed findings regarding its association with physical activity. Liangpunsakul et al.^[53]^ analyzed 10,550 participants from the National Health and Nutrition Examination Survey III and found that those who engaged in hazardous alcohol drinking (defined as > 14 drinks/wk in men and > 7 drink/wk in women) had significantly lower levels of physical activity compared with moderate drinkers and abstainers.^[53]^ Damian and Mendelson^[54]^ analyzed data from 4,828 adults and found that those who were categorized as physically active (responding “often” or “sometimes” to the question: “How often do you engage in sport/exercise?”) were more likely to be in remission from alcohol use problems [OR = 1.67 (95% CI = 1.29–2.17)] compared with physically inactive adults (answering “rarely” or “never”). Interestingly, there were no significant differences between the active and inactive groups on whether they had a current diagnosis of an alcohol use disorder [OR = 1.38 (95% CI = 0.86–2.22)].^[54]^ Conversely, French et al.^[55]^ analyzed data from 230,856 respondents from the Behavioral Risk Factor Surveillance System and found a positive association between alcohol use in the past 30 days and total weekly minutes of exercise time. This relationship persisted even among the very heavy drinkers (≥21 drinks/wk for women and ≥ 37 drinks/wk for men).^[55]^

There are longitudinal studies that have examined the relationship between physical activity and development of risky drinking or alcohol use disorders in adults. Härkönen et al.^[56]^ found that among 380 adults who were identified as “risky” drinkers at baseline (consuming ≥ 21 drinks/wk for men and ≥ 14 drinks/wk for women; excluding those with diagnosed alcohol use disorders), about half of these individuals (n = 185, 48.7%) remained risky drinkers 11 years later. The authors found that having a higher education, being male, and smoking at baseline significantly predicted risky drinking at follow-up, but baseline physical activity did not significantly predict or protect against risky drinking.^[56]^ Ejsing et al.^[57]^ analyzed data from 18,359 participants who were followed for an average of 21 years. Results indicated that the risk of developing an alcohol use disorder was significantly greater among participants who reported no leisure-time physical activity [HR = 1.45 (95% CI = 1.01–2.09) for women; HR = 1.64 (95% CI = 1.29–2.1) for men] compared with moderate-to-highly active men and women after adjusting for potential confounders including smoking and baseline alcohol intake. The relationship between physical inactivity and alcohol use disorder incidence remained even after stratifying analyses by low or high levels of alcohol consumption at baseline.^[57]^ Overall, there is still no clear relationship between physical activity and the development of problematic drinking or a clinical alcohol use disorder; however, there is a large body of evidence suggesting that physical activity is associated with moderate levels of alcohol consumption among adults, which is often regarded as a health promoting behavior.

## Older adults (approximately 55 years old and older)

The prevalence of SUDs in the older adult population is low compared with other age groups; however, the proportional admission rates into substance use treatment programs for older adults more than doubled between 2000 and 2012.^[58]^ Alcohol is the primary substance of abuse for older adults (64% of admissions), though the admission rates for other substances, including opioids and cannabis have increased sharply by 221% and 150% respectively among older adults. There are also concerning data suggesting that older adults develop SUDs without a history of abuse, because half of the individuals in treatment report that their initiation of substance use occurred after 25 years old.^[58]^ Older adults may be at an increased risk of substance use initiation because of the changes that occur with this developmental period such as having fewer daily responsibilities, transitioning to retirement, restructuring or loss of social relationships, and deteriorating physical and mental health, which may further reduce health-related quality of life and increase opportunities for medication misuse.^[24]^

No longitudinal studies have directly examined the effects of physical activity on the development of SUDs in older adults; however, healthy behaviors cluster together in older adults, and these patterns contribute to better physical and mental health with age. For example, Santini et al.^[59]^ determined that for adults 50 years and older, spending more time in a variety of activities (including physical activity and exercise) and having greater levels of social integration at baseline were associated with a reduced risk of problematic drinking 2 years later.^[59]^ Continued social integration in older adulthood also appears to be a part of good general health. Adults with higher levels of loneliness are more likely to be current smokers and are less likely to be physically active than adults who are not lonely.^[15]^ Loneliness among adults has also been associated with greater psychological distress and depression, both of which are comorbid with SUDs and inversely related to physical activity.^[60,61]^ Beyond these indirect associations of a healthy lifestyle with good physical and mental health, there is limited evidence surrounding the specific effects of increased physical activity and reduced substance use among older adults. Research focused on both prevention and treatment is urgently needed in this understudied, yet rapidly growing, subset of adults with SUDs. The current healthcare system is not equipped to treat SUDs in this booming patient population, particularly since the etiology and treatment of SUDs in older adults may vary considerably from the etiology and treatment of SUDs in adolescents and young adults. Table 1 depicts a summary of the possible relationships between physical activity and substance use in each life stage based on the current data.

**TABLE 1.**
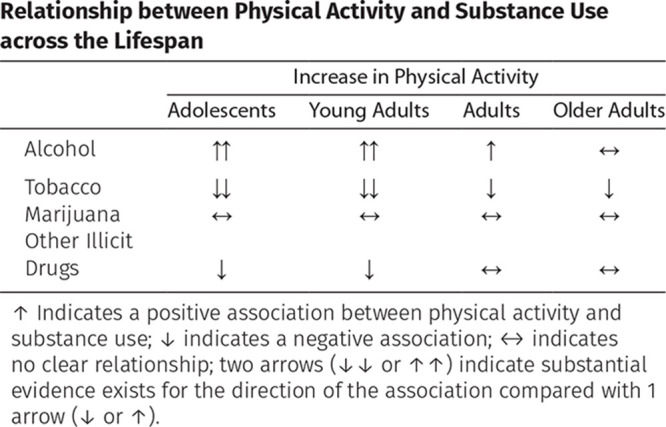
Relationship between Physical Activity and Substance Use across the Lifespan

## Limitations

There are several limitations in this area of research. First, no study in this review utilized objective measures of physical activity (eg, accelerometry). It is well known that subjective measures of physical activity are vulnerable to recall and self-report bias. Self-reported physical activity is typically an overestimate of actual physical activity, and its validity with objective measures is around 0.50 for adolescents and adults, depending on the tool used.^[62]^ Although objective measures of physical activity are increasingly utilized in observational studies, their cost, burden, and feasibility remain significant barriers to widespread use in large population-based research. Furthermore, validated subjective questionnaires, though less accurate, are still able to delineate relative amounts, type, and place of physical activity participation in a population-based sample, which can provide useful information from a public health perspective. Unfortunately, most studies in this review also did not use validated subjective measures of physical activity, instead often using a single question to assess physical activity frequency. Thus, the measurement of physical activity in this literature is tenuous, and any inferences made regarding the relationship between physical activity and substance use must consider this. Relatedly, the majority of the studies conducted in adolescents and young adults have assessed physical activity in the context of sports participation, which is a crude and complicated surrogate for actual exercise participation. There is substantial variation in the type and amount of physical activity between sports, and any findings are presumably confounded by the social dynamics of the sport. Many sports teams also have strict rules surrounding drug use, which may include testing for nicotine or other illicit substances but not necessarily for alcohol.

Along with indeterminate physical activity measurement, there was a wide range of measurement approaches and definitions for substance use, which encompassed lifetime use, regular use, at-risk use, heavy use, or a clinical endpoint such as an SUD. Most of the reviewed studies relied on self-report of substance use rather than objective measures (eg, toxicology screen) or clinical endpoints. Subjective measures of substance use are susceptible to bias for many reasons.^[63]^ Estimating the exact amount of substance use is challenging due to individual interpretations of what is considered a “standard” amount, how the drug is consumed (eg, ingested, intravenously), and/or concentrations of the psychoactive elements (eg, THC in marijuana or alcohol concentrations). Participants may be unwilling to report illegal use of substances (eg, underage use or illicit use) for fear of legal repercussions or because of social desirability bias. There is also a strong stigma that exists surrounding drug use, dependence, and treatment in many communities.

Even if accurate measurement of substance use could be guaranteed, it is also important to note that general substance use does not always lead to substance abuse or an SUD. In fact, the majority of individuals who experiment with drugs and alcohol do not develop an SUD.^[64]^ Thus, it is necessary to examine physical activity as 1 of many vulnerability or protective factors influencing whether an individual will escalate from drug experimentation to a clinical SUD. In addition, many of the reviewed studies were cross-sectional, indicating a possible reverse causality that substance use might prevent physical activity participation just as easily as physical activity might help prevent substance use. It is also possible that physical activity and substance use are simply behavioral tradeoffs (ie, spending time in 1 behavior (exercise) diminishes the amount of time you can spend in another behavior (eg, substance use)). Beyond being behaviorally incompatible, it is not clear at this time whether physical activity elicits other protective adaptations against drug use vulnerability because few studies have assessed potential mechanisms such as social-cognitive development, self-esteem, or other neurobiological effects. Finally, there are no randomized, controlled exercise trials examining exercise’s effects on later incidence of SUDs, likely due to the cost and sample size that would be required for such a study. There is a growing body of research suggesting that exercise may be effective in the treatment of SUDs and prevention of relapse,^[65,66]^ but findings so far have been limited due to small sample sizes with high dropout rates, and mixed results across different substances of abuse and interventions.^[19,67]^

## Conclusions and future directions

Physical activity (ie, predominantly sports participation) during adolescence and young adulthood—the developmental periods with the highest rates of substance use initiation—is protective against tobacco use and illicit drug use (except marijuana). Unfortunately, physical activity during these periods also appears to be associated with increased alcohol use possibly due to social factors related to team sports. These relationships seem to persist into later life, with lower rates of tobacco use and higher rates of alcohol use among physically active adults. There is not enough evidence at this time to state whether physical activity in adulthood reduces the risk of future illicit drug use or clinical SUDs, such as alcohol use disorder. Additional longitudinal research using objective measures of physical activity along with clinically defined SUD outcomes is warranted across all age groups. Observational studies should also include potential mediators in their analyses to help clarify conflicting findings across studies. Including a quantifiable exposure (physical activity), a diagnosable outcome (SUD), and a plausible mechanistic pathway between them will elucidate the potential efficacy of physical activity in the prevention and treatment of SUDs. The results from these investigations are needed to inform the design of future randomized controlled trials (RCTs), which will be able to determine causality. Data from RCTs involving a physical activity intervention on incident SUDs are limited at this time, potentially due to the magnitude, cost, and duration that would be required for such a trial. However, through identification and thorough investigation of the potential mechanisms underlying physical activity and substance use, it is possible to design an RCT in a vulnerable population (eg, adolescents) wherein the primary outcome is not necessarily a diagnosable SUD but rather meaningful changes in predictors of SUDs (eg, rates of use, self-efficacy, social integration, depression). It is then possible to track these adolescents into young adulthood to determine if the intervention affected later physical activity levels and incident SUDs.

The importance of physical activity and sports participation on physical and mental health during adolescence and young adulthood (and throughout life) cannot be overstated. While the current findings suggest that physical activity is associated with alcohol use, this does not imply that parents, school counselors, or clinicians should advise youth to avoid sports or exercise. Instead, these findings suggest that more thorough education, identification, and monitoring of alcohol and substance use among sports participants is warranted. Individuals should be able to enjoy the numerous benefits of physical activity, including reduced tobacco and drug use, while having the resources to avoid negative health behaviors (eg, alcohol use).

## Acknowledgments

DC Lee was funded by the National Institutes of Health (HL133069). The content is solely the responsibility of the authors and does not necessarily represent the official views of the National Institutes of Health.

## Disclosure

The authors have no financial interest to declare in relation to the content of this article. The Article Processing Charge paid for by Progress in Preventive Medicine at the discretion of the Editor-in-Chief.
